# Hybrid-abutment-restoration: effect of material type on torque maintenance and fracture resistance after thermal aging

**DOI:** 10.1186/s40729-020-00220-y

**Published:** 2020-06-24

**Authors:** Walid Al-Zordk, Ahmed Elmisery, Mohamed Ghazy

**Affiliations:** Department of Fixed Prosthodontics, Mansoura University, PO Box 35516, Mansoura, Dakahlia Egypt

**Keywords:** Implant, Non-segmented, Torque loss, Fracture resistance, Titanium base

## Abstract

**Purpose:**

To evaluate the tightening torque maintenance with zirconia, lithium disilicate, and polyetheretherketone (PEEK) hybrid-abutment-crowns after thermal aging, in addition to assess the fracture resistance of hybrid-abutment-crowns fabricated with different materials.

**Materials and methods:**

Thirty implants were restored with identical hybrid-abutment-crowns, resembling the maxillary first premolar, fabricated from zirconia (Zr), lithium disilicate (L2), or ceramic-reinforced PEEK (PE). The three groups (*n* = 10) were constructed utilizing a Ti-base. After bonding, each restoration was secured in its respective implant with a torque of 25 Ncm. All restorations were subjected to thermal aging for 7000 cycles. The loosening torque was assessed utilizing the digital torque meter. Each restoration was subjected to fracture testing and the mode of failure was determined.

**Results:**

Zr group displayed the highest mean torque loss value (2.70 ± 0.59 Ncm) with the mean loosening torque value of 22.38 ± 0.68 Ncm. PE group displayed the lowest mean torque loss (2.55 ± 0.50 Ncm) with mean loosening torque value of 22.61 ± 0.59 Ncm. There was no significant difference between study groups regarding loosening torque (*p* = 0.68), torque loss (*p* = 0.80), and percentage of torque loss (*p* = 0.79). There was significant difference regarding the mean fracture load value between Zr and PE groups. However, there was no significant difference (*p* = 0.05) regarding mean fracture load value between L2 and PE groups.

**Conclusion:**

The hybrid-abutment-crown material does not affect the torque maintenance after thermal aging. Based on fracture load, zirconia hybrid-abutment-crown can be used, while lithium disilicate and PEEK hybrid-abutment-crowns may cautiously serve in premolar region.

## Introduction

Using osseointegrated dental implants for replacement of missing teeth in esthetic region has become a treatment modality to restore both function and esthetics with high rate of survival [1, 2]. It is important to harmonize the implant-supported restoration with the surrounding soft tissue and adjacent natural teeth [3]. Selecting the proper abutment is essential for achievement of a mechanically settled and esthetically pleasant restoration. The prefabricated abutments have several advantages such as simple utilization and inexpensive. However, these abutments rarely offer proper form and shape with the main problem reported is the platform diameter and associated emergence profile [4].

The custom abutments are required in specific clinical situations such as when the collar height needed is not offered by the implant manufacturers, reproduction of the original cross-sectional profile of the tooth to obtain an ideal emergence profile, and insufficient inter-occlusal space for the restoration [5]. With cast custom abutments, the alloys used in the casting process are subject to numerous factors which eventually make the characteristic features of the eventual abutment unpredictable. However, milled custom abutments are fabricated with better quality, superior strength, and durability. During the milling process of CAD̸CAM-fabricated custom abutments, the materials are not liable to the casting process that can cause dimensional inaccuracy and improper fit [6]. There are two possibilities for esthetic custom abutment: as hybrid-abutment-crown with the abutment and crown are produced as single piece which is attached to Ti-base, or as a hybrid abutment attached to Ti-base with a separated crown [3, 7–10].

One of the most critical mechanical complications is the loosening of abutment or prosthesis screw. Currently, the incidence of screw loosening extends between 7 to 11% [11]. Screw loosening can cause unbalanced distribution of occlusal forces, screw and implant fracture, micro-gab space between abutment, and implant that can allow bacterial ingress that will affect the osseointegration. Screw loosening can be attributed to variety of factors such as insufficient tightening force, improper placement of the implant, excess mechanical loads than normal, and changes in temperature in the oral cavity [5, 12]. A study examined the stability of implant protheses with stock abutment, cast gold abutment, and milled custom abutment, and reported that the type of the abutment did not possess a significant influence on screw loosening after dynamic loading [13]. Another study showed good screw joint stability for stock and custom titanium abutments after cyclic loading [14].

The implant abutment must be fabricated from materials which are biocompatible with sufficient mechanical properties to perform functional, esthetic, and biological demands [4]. Additionally, it should passively and accurately fit with its corresponding mating implants to lessen complications such as abutment fractures and screw loosening. A wide assortment of abutment materials is available on the dental market. Elsayed et al. [7] studied the effect of crown materials on fracture strength of custom titanium and zirconia abutments and concluded that all tested abutment and crown materials could be deemed to possess fracture strengths appropriate for clinical implementation.

Ceramic restorations are susceptible to slow crack growth at the tip of the surface flaws exposed to a moisture environment as a result of hydrolysis of the silicate bonds [15]. Thermal changes and the testing of the fracture resistance of ceramic restorations could provide a better understanding of the clinical outcomes [16]. The thermal aging has been proposed to simulate the extreme conditions commonly experienced in the oral environment [8, 10, 17]. The thermal aging could induce the degradation of resin cement at the titanium base insert and the ceramic interface that may affect the load transfer at the interface [18, 19]. A major challenge today is understanding the response to each restorative material. Therefore, the aim of this study was to investigate the effect of thermal aging on torque maintenance of zirconia, lithium disilicate, and PEEK hybrid-abutment-crowns. Also, the fracture resistance of zirconia, lithium disilicate, and PEEK hybrid-abutment-crowns was studied. The tested null hypothesis was that no difference in torque loss of zirconia, lithium disilicate, and PEEK hybrid-abutment-restorations after thermal cycling. Second null hypothesis was that there is a difference in fracture resistance of zirconia, lithium disilicate, and PEEK hybrid-abutment-crowns.

## Materials and methods

A total of thirty titanium implants with its corresponding titanium bases were used in the current study. Each implant was restored with an identical non-segmented hybrid-abutment-crown restoration resembling the maxillary first premolar fabricated from zirconia (Z-CAD, Metoxit, Switzerland), lithium disilicate (IPS e.max CAD, Ivoclar Vivadent, Liechtenstien), or ceramic-reinforced PEEK (Bredent, Senden, Germany). The groups were designed as follow (*n* = 10): zirconia hybrid-abutment-crown restoration (Zr), lithium disilicate hybrid-abutment-crown restoration (L2), and ceramic-reinforced PEEK hybrid-abutment-crown restoration (PE).

The first premolar tooth was removed from maxillary model (M-1560, Colombia Dentoform Corp., New York) to mimic missed maxillary first premolar tooth. A silicone mold was fabricated using silicone duplicating material (Replisil 22 N, Dent-e-con, Germany), which was used to fabricate thirty epoxy casts. For proper osteotomy preparation and standardization of fixture angulation, a surgical guide was designed (exocad, exoplan, Germany) and 3-D printed (Zenith 3-D printer, Korea). After preparation of the osteotomy, each epoxy cast received a fixture of 4-mm diameter and 10-mm length (Implantium, Dentium Co, Seoul, Korea), and having internal connection. The fixtures were installed to the level of the first thread. The titanium bases (Dentium Custom Abutment, Dentium Co., Seoul, Korea) were 3 mm in height. Each titanium base was aligned over its corresponding fixture and anchored with screw driver.

Epoxy casts and titanium bases were sprayed by antireflection scan powder (Telescan, DFS Diamon, Germany). Then, each titanium base was scanned (DOF, 3D Scanner Swing, Korea). Single-unit crown was designed using the CAD software (exocad Dental DB software, Germany) to accommodate the anatomy and average dimensions of maxillary first premolar crown. Each restoration was virtually seated on its corresponding titanium base and the crew channel was determined. The zirconia restorations were milled out of zirconia CAD̸CAM blocks (Z-CAD, Metoxit, LOT V17292), lithium disilicate restorations were milled out of glass-ceramic blocks (IPS e.max CAD, Ivoclar Vivadent, LOT 0030223), and PEEK restorations were milled out of ceramic-reinforced blocks (BioHPP, Bredent, LOT 467881). Zirconia restorations were sintered in zirconia sintering furnace (SinterMax Model T1700, SinterMax, USA) according to manufacturer’s instructions. Lithium disilicate restorations were subjected to crystallization firing in porcelain furnace (EP500 Programat, Ivoclar Vivadent) according to manufacturer’s instructions. Each restoration was seated to its corresponding titanium base prior to bonding to test the accuracy of fit.

Each titanium base was airborne-particle abraded using 110-μm aluminum oxide at 2-bar pressure for 10 s according to manufacturer’s instructions, then ultrasonically cleaned for 10 min. The titanium bases were dried and primed (MKZ Primer, Bredent, LOT 439150). For zirconia restorations, the inner surfaces of were airborne-particle abraded using 50-μm aluminum oxide at pressure of 2-bar pressure for 10 s according to manufacturer’s instructions, then ultrasonically cleaned for 10 min. The inner surfaces of zirconia restorations were dried and primed (MKZ Primer, Bredent). The inner surfaces of lithium disilicate restorations were treated with 5% hydrofluoric acid (Ceramic Etchant, Dentobond, France) for 60 s according to manufacturer’s instructions, followed by ultrasonic cleaning in for 10 min. Then, the surfaces of lithium disilicate restorations were dried and primed (MKZ Primer, Bredent). With PEEK, the inner surfaces were airborne-particle abraded with 50-μm aluminum oxide at 2-bar pressure for 10 s according to manufacturer’s instructions, followed by ultrasonic cleaning in distilled water for 10 min. Then, the surfaces were dried and primed (Visio-Link Primer, Bredent, LOT 153141).

Each restoration was cemented to its corresponding titanium base using adhesive resin cement (DTK Adhesive, Bredent, LOT 476249) according to manufactures’ recommendations. Each hybrid-abutment-crown was torqued to respective implant via abutment titanium screws at 25 Ncm following the manufacturer’s instructions with a torque control system (TSD-50 Torque Screw Driver S/N17000060, Electromatic Equip`t Co., NY, USA), and retightened after 10 min to decrease settling effect [3]. The screw access channels were sealed, and all the bonded specimens were stored in distilled water for 3 days to guarantee complete setting of the adhesive cements.

### Thermal aging

All specimens were subjected to artificial thermal aging in the thermocycler (SD Mechatronic Thermocycler, Germany). The thermocycling was performed for 7000 cycles that corresponds to 2 years of clinical function in 5–55 °C water bath with 30 s dwell time and 5 s transfer time [16].

### Torque loss

After thermal aging, the loosening torque was measured in a counter-clockwise movement using the digital torque meter (TSD Digital Torque Screw Driver, Electromatic Equipt Co., USA) and the data was collected for calculations of the torque loss.

### Fracture testing

Each specimen was loaded to failure by vertical compressive load using universal testing machine (Nexygen Lodel LRX-plus, Lioyd Ltd.). Force was applied with a crosshead speed of 0.5 mm/min using steel rod with diameter of 6 mm. The failure load (*N*) was recorded.

### Statistical analysis

Data was statistically resolved utilizing the Statistical Package for Social Science software (SPSS v23, SPSS Inc., Chicago IL, USA). For the assessment of the normality and equality of variance, data was analyzed with Kolmogorov-Simirnov test and Levene test. One-way analysis of variance (ANOVA) followed by the post hoc Tukey test were employed for comparing data. The statistical significance level was set at *p* ≤ 0.05.

## Results

### Loosening torque, torque loss, and percentage of torque loss

**Fig. 1:**
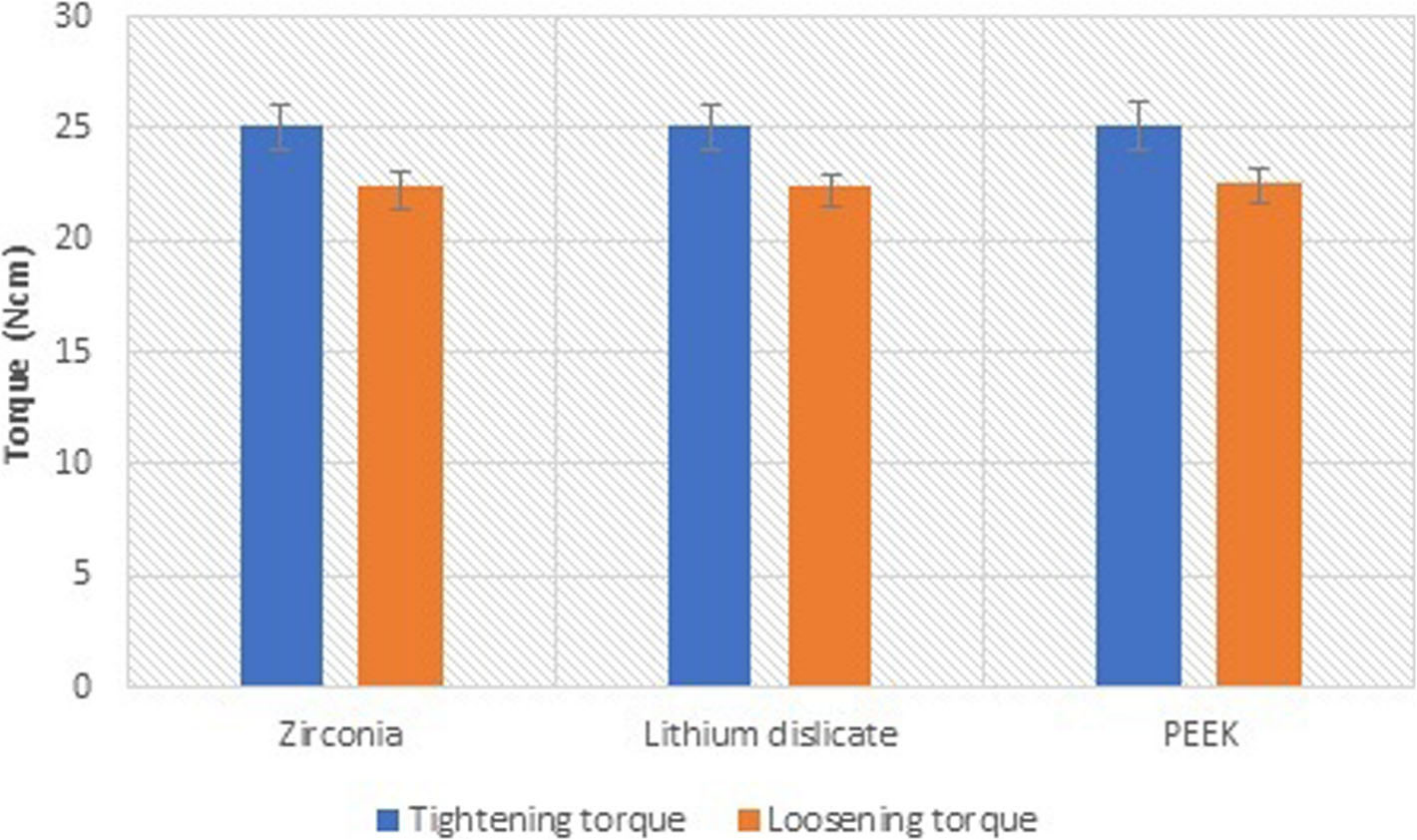
The tightening torque and the loosening torque of zirconia and lithium disilicate and PEEK groups

Zirconia hybrid-abutment-crown restorations recorded the highest mean torque loss value (2.70 ± 0.59 Ncm), with the mean loosening torque value of 22.38 ± 0.68 Ncm. For lithium disilicate hybrid-abutment-crown restorations, the mean loosening torque value was 2.63 ± 0.46 Ncm, and the mean loosening torque value was 22.49 ± 0.47 Ncm. PEEK hybrid-abutment-crown restorations showed the lowest mean torque loss value (2.55 ± 0.50 Ncm) with mean loosening torque value of 22.61 ± 0.59 Ncm. There was no significant difference between study groups regarding loosening torque (*p* = 0.68), torque loss (*p* = 0.80), and percentage of torque loss (*p* = 0.79) (Table 1). Within each group, there was significant difference between the mean tightening torque and the mean loosening torque for zirconia group (*p* ˂ .001), lithium disilicate group (*p* ˂ .001), and PEEK group (*p* ˂ .001) (Fig 1).

**Table 1:** Comparison between zirconia, lithium disilicate, and PEEK hybrid-abutment-restorations regarding tightening torque (Ncm), loosening torque (Ncm), torque loss (Ncm), and percentage of torque loss (%)

	Zirconia	Lithium disilicate	PEEK	*p* value
Tightening torque	25.08 ± 0.13	25.12 ± 0.15	25.14 ± 0.19	0.70
Loosening torque	22.38 ± 0.68	22.49 ± 0.47	22.61 ± 0.59	0.68
Torque loss	2.70 ± 0.59	2.63 ± 0.46	2.55 ± 0.50	0.80
Percentage of torque loss	10.74 ± 2.39	10.45 ± 1.83	10.11 ± 1.99	0.79

### Fracture load

Zirconia hybrid-abutment-crown restorations showed the highest mean maximum fracture load value (1567.17 ± 111.39 N) followed by PEEK hybrid-abutment-crown restorations (556.76 ± 95.32 N), while lithium disilicate hybrid-abutment-crown restorations showed the smallest mean fracture load value (460.26 ± 43.08 N). Post hoc Tukey test showed that there was significant difference (*p* < 0.001) regarding the mean fracture load value between zirconia hybrid-abutment-crown restorations and PEEK hybrid-abutment-crown restorations, and there was significant difference (*p* < 0.001) regarding mean fracture load value between lithium disilicate hybrid-abutment-crown restorations and zirconia hybrid-abutment-crown restorations (Table 2). However, there was no statistical significant difference (*p* = 0.05) regarding mean fracture load value between lithium disilicate hybrid-abutment-crown restorations and PEEK hybrid-abutment-crown restorations.

**Table 2:** Mean and standard deviations of fracture resistance values (*n*) of experimental groups

Restoration	Fracture load (*n*)
Zirconia	1567.17 ± 111.39^a b^
Lithium disilicate	460.26 ± 43.08^b A^
PEEK	556.76 ± 95.32^a A^

Lower case similar letters indicate significant difference (*p* ˂ .05) while upper case similar letters indicate no significant difference (*p* ˃ .05)

### Failure mode

**Fig. 2:**
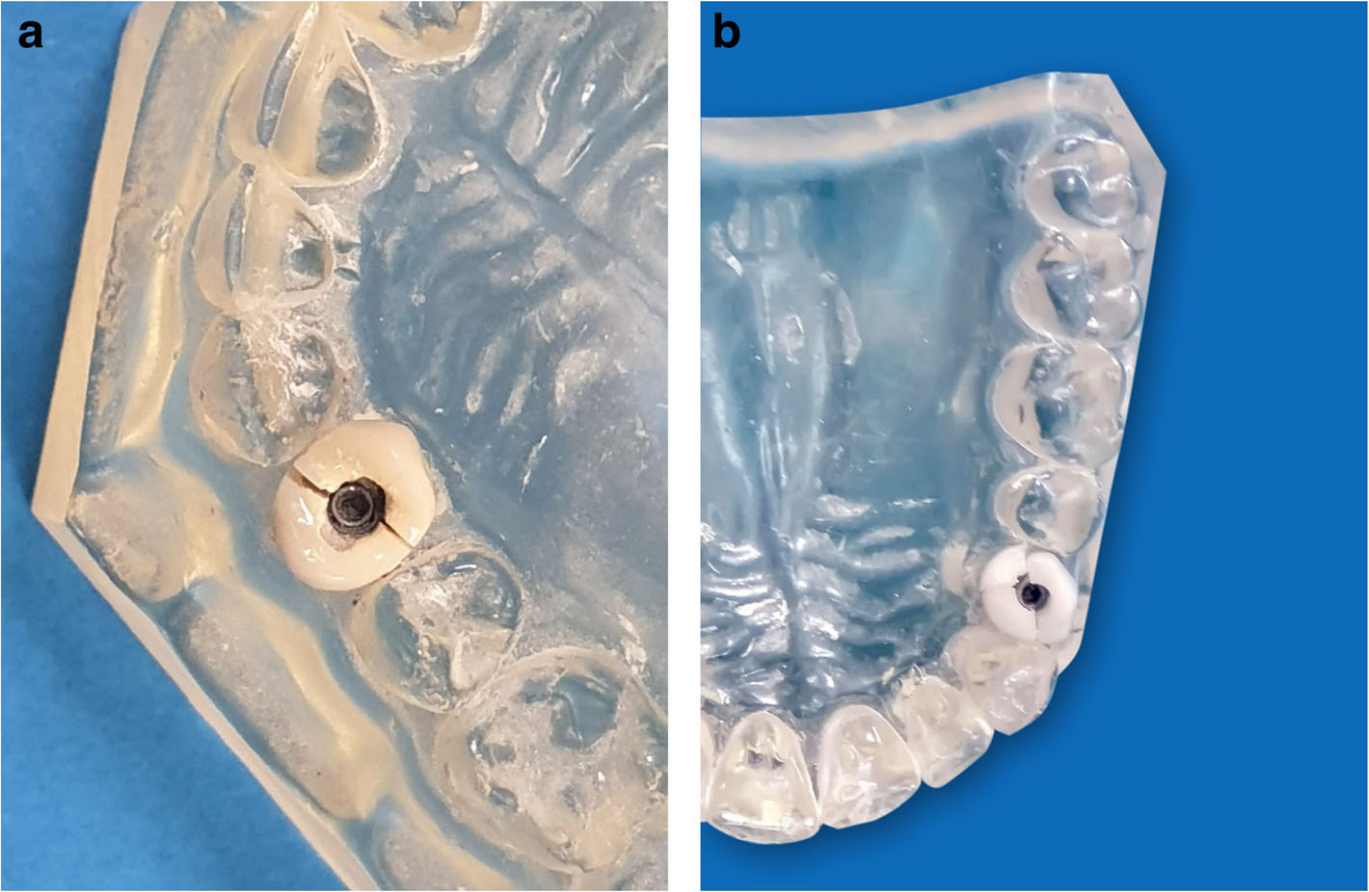
Failure modes of hybrid-abutment-restorations. **a** The fracture was either two halves, **b** or three fragments

After fracture load testing, digital photos were taken for each specimen to determine the failure modes (Fig. 2). All zirconia hybrid-abutment-crown restorations showed complete vertical fracture into two halves above the shoulder of the titanium base. For lithium disilicate, seven restorations showed complete vertical fracture into two halves above the shoulder of the titanium base and three restorations showed fracture into three fragments. As for PEEK, all restorations showed complete vertical fracture above the shoulder of the titanium base.

## Discussion

The current study focused on posterior hybrid-abutment-crown manufactured from zirconia, lithium disilicate, and PEEK. The torque maintenance and fracture loads were studied. The findings of the present study confirm the first part of stated working hypotheses that no difference in torque loss of zirconia, lithium disilicate, and PEEK hybrid-abutment-crowns after thermal cycling. Also, the second null hypothesis that the material would have an influence on fracture resistance was confirmed.

An important concern in implant prosthodontics is the technique of attachment between the prosthetic abutment (or the fixture) using either retaining screw or cementation. Cement-retained restoration was emerged to deal with the functional and esthetic requirements of screw-retained restoration and the issue of less-optimal fixture angulation. However, the concept of cement-retained restoration has the disadvantage of making excess cement removal critical. Prevalence of peri-implant diseases ranged between 1.9 and 75% of implant-supported restorations employing cement-retained concept, with percentage ranged between 33 to 100% correlated with excess cement [20]. With screw-retained hybrid-abutment designs, the restoration can be cemented extra-orally on the titanium base and excess cement can be accomplished with ease. In the present study, hybrid-abutment-crown design was tested. Roberts et al. [21] studied the fracture resistance of hybrid-abutment-crowns and hybrid-abutments with separate crowns, and concluded that the hybrid-abutment-crowns showed the highest fracture resistance. Also, they reported that separating the abutment and crown inherently produces a weaker overall prosthetic restoration than fabricating a full-contour abutment-crown design. The use of hybrid-abutment-crown design can combine some advantages of both screw-retained and cement-retained concepts by eliminating some of problems of both. Nevertheless, the demand for an optimal surgical implant placement is requested [3].

The abutment material could influence the joint stability [5]. In the present study, torque maintenance was tested using digital torque meter device [22–24]. There were no significant differences regarding the mean torque loss values between the three groups. This result was in agreement with other studies [22, 25]. In the present study, there were significant differences between the tightening torque and loosening torque, within each group. The thermal aging may have affected the integrity of the resin cement with adverse effect on load transfer [18, 19]. Tzannas et al. [26] reported that the removal torque is 85 up to 90% of tightening torque. However, Paek et al. [14] reported that there was no significant difference among initial tightening torque first or second removal torque. Their results may be attributed to the employment of two different devices for screw tightening in their study.

Maximum masticatory forces reported in premolar teeth were in the range of 200–445 N, whereas, the maximum masticatory forces in molar teeth were about 900 N [27, 28]. Additionally, parafunctional disorders (such as bruxism) can produce higher bite forces. Masticatory forces of these ranges were sustained and exceeded by restorations of zirconia group only. Therefore, it is not recommended to use lithium disilicate and PEEK hybrid-abutment-restorations in posterior molar region [10]. The fracture resistance of zirconia restoration was higher than lithium disilicate and PEEK restorations, which is related to specific material properties [29, 30]. The present findings are consistent with the results of Honda et al. [9], who investigated the fracture loads of hybrid-abutment-crown fabricated with different restorative materials. Elshiyab et al. [8] reported a fracture resistance of monolayer zirconia crowns cemented to hybrid abutments of 3929 N. Their results may be explained by using larger diameter implant (5.5 mm) and 4-mm high titanium base. With bonded titanium base, the wear at implant-abutment connection and fracture properties of zirconia abutments were improved [5, 9]. The potential weakness of ceramic discontinuity of screw access hole is minimized with the application of stronger ceramics such as zirconia [4]. Although the high fracture load, zirconia is a brittle material and cannot bear tension. Additionally, its long-term stability may be limited because of low-temperature degradation [1]. Based on the results of the current study, the zirconia hybrid-abutment-crowns would properly have enough mechanical strength clinically. The excellent optical properties, good mechanical characteristics, and the biocompatibility of lithium disilicate material raised the chance of using lithium disilicate ceramic with titanium base [3, 22]. PEEK material has a stiffness double that of lithium disilicate and reduce the stresses directed on implant [31]. Compared with zirconia customized abutment, Kaleli et al. [32] studied the biomechanical behavior of PEEK and showed that the PEEK-customized abutment demonstrated low stresses within the implant-abutment complex but also showed elevated stresses in the prosthetic crown and the titanium base.

The current results revealed that fracture evolved about the occlusal access area. This may be attributed to the existence of an occlusal access hole disturbs the structural integrity of the restoration and increase the tension, with stress peaks laterally in the occlusal area [9]. Additionally, the limited area of titanium base available for bonding may play a contributing role in current fracture modes. Thus, it is advised to investigate the influence of the height of the titanium base on the fractures of hybrid-abutment-restorations [10]. In the present study, no damage or plastic deformation was observed on the titanium base, in the abutment screw, at implant-base connection, or in the fixture. These results are consistent with findings of other researches [3, 33]. Alshhaf et al. [34] reported that the titanium base with zirconia abutment functions as a replacement for the weakest area of the zirconia abutment. Therefore, the titanium base can reinforce the fracture strength of a zirconia abutment [4, 34].

This study has limitations, such as the hybrid-abutment-crown restorations tested did not undergo physiologic fatigue loading. Thus, further studies should investigate the effects of cyclic loading in a wet environment to simulate intra-oral condition. Also, additional researches are needed to completely recognize the variables that can induce loss of torque values in hybrid-abutment-restorations. Additional long-term clinical investigations will be needed to set up the clinical relevance of our results and to determine the in vivo performance of hybrid-abutment-restorations.

## Conclusions

Within the limitations of the current investigation, the following could be concluded:

1. The hybrid-abutment-crown material (zirconia, lithium disilicate, and PEEK) does not affect the torque maintenance after thermal aging.
2. Based on fracture load, zirconia hybrid-abutment-crown can be used, while lithium disilicate and PEEK hybrid-abutment-crowns may cautiously serve in premolar region.

## Data Availability

All data and materials are available from the corresponding author.

## Abbreviations

PEEK: Polyetheretherketone
Zr: Zirconia
L2: Lithium disilicate
PE: Ceramic-reinforced PEEK
CAD̸CAM: Computer aided design-computer aided manufacturing
ANOVA: One-way analysis of variance
